# Impact of population density and weather on COVID-19 pandemic and SARS-CoV-2 mutation frequency in Bangladesh

**DOI:** 10.1017/S0950268821000029

**Published:** 2021-01-07

**Authors:** Nadim Sharif, Shuvra Kanti Dey

**Affiliations:** Department of Microbiology, Jahangirnagar University, Savar, Dhaka 1342, Bangladesh

**Keywords:** Bangladesh, COVID-19, mutation frequency, population density, temperature, UV index

## Abstract

Coronavirus disease-2019 (COVID-19) has caused the recent pandemic worldwide. Research studies are focused on various factors affecting the pandemic to find effective vaccine or therapeutics against COVID-19. Environmental factors are the important regulators of COVID-19 pandemic. This study aims to determine the impact of weather on the COVID-19 cases, fatalities and frequency of mutations in Bangladesh. The impacts were determined on 1, 7 and 14 days of the case. The study was conducted based on Spearman's correlation coefficients. The highest correlation was found between population density and cases (*r*_s_ = 0.712). Among metrological parameters, average temperature had the strongest correlation (*r*_s_ = −0.675) with the cases. About 82% of Bangladeshi isolates had D614G at spike protein. Both temperature and UV index had strong effects on the frequency of mutations. Among host factors, coinfection is highly associated with frequency of different mutations. This study will give a complete picture of the effects of metrological parameters on COVID-19 cases, fatalities and mutation frequency that will help the authorities to take proper decisions.

## Introduction

Severe acute respiratory syndrome coronavirus-2 (SARS-CoV-2) (family − *Coronaviridae*) has triggered the coronavirus 2019 (COVID-19) pandemic worldwide [1, 2]. Other viruses of this family − severe acute respiratory syndrome (SARS) virus and Middle East severe acute respiratory syndrome (MERS) virus have caused local outbreaks in the past [3]. Primarily, coronaviruses can infect the upper respiratory system in humans [1–3]. First, two isolates − human coronavirus 229E and human coronavirus OC43 were detected in the early 1960s in patients with pneumonia-like symptoms [1, 3]. As of 12 August 2020, COVID-19 has infected 19 936 210 individuals and caused the death of 732 499 infected persons in 220 countries and territories worldwide [4]. Currently, COVID-19 is one of the major health burdens worldwide [5, 6].

COVID-19 infected patients develop various clinical symptoms, as the virus infects the upper respiratory tract [1]. Among the most common clinical features, fever, cough, sore throat, shortness of breath are significant. Most of the patients (70%) develop mild symptoms. Asymptomatic patients and patients with mild symptom have good recovery rate [1, 4, 7]. However, other clinical features such as chill, feelings of shaking, loss of taste or smell, headache, rash and muscle pain have been diagnosed in numerous COVID-19 patients worldwide. In severe cases, acute pneumonia, acute respiratory syndrome, kidney failure, difficulty in breathing and failure of multiple organs have been reported [1, 8]. Comorbidities are associated with significant frequency of the COVID-19 fatalities worldwide [1, 4, 9].

Emergence and reemergence of infectious viruses have been mostly reported during winter with cold temperature worldwide [10]. Seasonality of different viruses such as West Nile viruses, rotavirus, adenovirus and many other viruses are well established now [11–13]. Different environmental factors − temperature, humidity, rainfall, UV intensity, wind speed etc. have played significant roles in COVID-19 pandemic [14–16]. Environmental factors occupy important role in the survival, viability and transmission of infectious viruses [17–22]. Some well-established modes of transmission for COVID-19 are contact (both direct and indirect), droplet nuclei and fomite (non-living objects) transmission [17–19]. Environmental factors can regulate the transmission by affecting the viability of coronaviruses in droplet nuclei and fomite [14, 20–23]. Besides, severity and expansion of the COVID-19 pandemic are affected by other factors like population density, hygienic conditions of mass people, duration of strict lock down and transportation of infected persons across borders [24].

In Bangladesh, the first COVID-19 case was detected on 07 March 2020. As of 10 August 2020, about 260 507 cases and 3438 fatalities due to COVID-19 have been reported in Bangladesh [25]. The total population of Bangladesh is about 170 306 468 with a population density of 1265 person/km^2^ [26]. COVID-19 cases and fatalities have been reported from 64 districts in Bangladesh. As of 10 August 2020, most of the COVID-19 cases were reported from Dhaka (60%), Chattogram (10%), Bogura (5%), Khulna (5%), Sylhet (5%) and Mymensingh (4%) in Bangladesh. As of 16 August 2020, about 20% of the total COVID-19 tests (1 364 189) were positive in Bangladesh. The case fatality rate of COVID-19 is about 1.3% in Bangladesh, which is higher (3.5%) in other countries worldwide [25].

SARS-CoV-2 is a positive sense virus with a non-segmented, single-stranded RNA (ssRNA) genome of ~30 000 bases [1, 2]. The genome can act as a direct mRNA as it contains a 5′ cap structure along with a 3′ poly (A) tail. Open reading frames (ORFs), 1a, 1b, 3a, 3b, 6, 7a, 7b, 8a, 8b and 9b are most common in SARS-CoV-2 genome. First two ORFs-1ab comprise of ~20 000 bases (two-third of genome) and encode for replicase proteins (non-structural proteins). Sixteen non-structural proteins (nsps) – nsp1 to nsp16 have been identified. Later ORFs (~10 000 bases) encode for four major structural proteins-spike (S), envelope (E), membrane (M) and nucleocapsid (N) [1, 2, 9]. The mutation frequency is moderate in coronavirus. Various factors including host and environmental factors are involved in the mutational events of coronaviruses [27, 28]. Mutations at S protein can change the pathogenesis of the virus and initiation of specific host immune responses [29]. Further, environmental factors such as high temperature, UV radiation and high humidity play active role in evolving mutant viruses [27]. Host factors including age, gender, immune status and presence of coinfections are also involved in mutation events [30].

The main aim of this study is to analyse the correlation between metrological factors and frequency of mutations in SARS-CoV-2. The second objective of this study is to determine the relationship between host factors and SARS-CoV-2 mutation frequency. The third objective of this study is to investigate the correlation between environmental factors and COVID-19 pandemic in Bangladesh. This study will provide a better insight on the effects of environmental factors on COVID-19 pandemic in Bangladesh.

## Materials and methods

### Study areas and duration

This study focused on the correlation of COVID-19 with metrological parameters in eight cities in Bangladesh. This study included Dhaka (23.71°N to 90.41°E), Chattogram (22°20′06″N to 91°49′57″E), Bogura (24°51′N to 89°22′E), Khulna (22°49′N to 89°33′E), Sylhet (24°54′N to 91°52′E) and Mymensingh (24°45′14″N to 90°24′11″E), Barishal (22°48′0″N to 90°30′0″E) and Rangpur (25°44′N to 89°15′E). With a population density of 46 997 person/km^2^, Dhaka is the most populous capital in the world. The study period was from 07 March 2020 to 14 August 2020. On 07 March 2020, COVID-19 patients were detected for the first time in Bangladesh and considered as day 1 for the study.

### Study data

The data of COVID-19 cases and fatalities were collected from official websites of the Directorate General of Health Services (DGHS) (https://covid19bd.idare.io/) and Institute of Epidemiology, Disease Control and Research (https://www.iedcr.gov.bd/website/) in Bangladesh, and cross-confirmed by analysing the data from official websites of WHO (www.who.int), Bing (www.bing.com/covid), Worldometers (www.worldometers.info/coronavirus/) and Johns Hopkins University (https://coronavirus.jhu.edu/). Environmental data including minimum temperature (⁰C), average temperature (°C), maximum temperature (°C), UV index, wind speed (km/h), rain fall (mm), relative humidity (%) were collected from different databases including official website of Bangladesh Meteorological Department (http://live4.bmd.gov.bd/satelite/v/sat_infrared/), meteoblue (www.meteoblue.com), AccuWeather (www.accuweather.com) and WeatherOnline (www.weatheronline.co.uk) during this study. The whole genome of SARS-CoV-2 isolated from Bangladesh and reference genome sequences were collected from the official website of GISAID (https://www.gisaid.org/). Accession number of sequences are: EPI_ISL_437912, EPI_ISL_445213, EPI_ISL_445214, EPI_ISL_445215, EPI_ISL_445216, EPI_ISL_445217, EPI_ISL_445244, EPI_ISL_447590, EPI_ISL_447897, EPI_ISL_447899, EPI_ISL_447904, EPI_ISL_450339, EPI_ISL_450340, EPI_ISL_450340, EPI_ISL_450341, EPI_ISL_450342, EPI_ISL_450343, EPI_ISL_450344, EPI_ISL_450345, EPI_ISL_450839, EPI_ISL_450840, EPI_ISL_450841, EPI_ISL_450842, EPI_ISL_450843, EPI_ISL_455420, EPI_ISL_455458, EPI_ISL_455459, EPI_ISL_458133, EPI_ISL_462090, EPI_ISL_462091, EPI_ISL_462092, EPI_ISL_462093, EPI_ISL_462094, EPI_ISL_462095, EPI_ISL_462096, EPI_ISL_462097, EPI_ISL_462098, EPI_ISL_464159, EPI_ISL_464160, EPI_ISL_464161, EPI_ISL_464162, EPI_ISL_464163, EPI_ISL_464164, EPI_ISL_466626, EPI_ISL_466627, EPI_ISL_466628, EPI_ISL_466629, EPI_ISL_466630, EPI_ISL_466636, EPI_ISL_466637, EPI_ISL_466638, EPI_ISL_466639, EPI_ISL_466644, EPI_ISL_466645, EPI_ISL_466649, EPI_ISL_466650, EPI_ISL_466686, EPI_ISL_466687, EPI_ISL_466688, EPI_ISL_466689, EPI_ISL_466690, EPI_ISL_466691, EPI_ISL_466692, EPI_ISL_466693, EPI_ISL_466694, EPI_ISL_468070-EPI_ISL_468078, EPI_ISL_469285, EPI_ISL_469286, EPI_ISL_469297-EPI_ISL_469300, EPI_ISL_470801, EPI_ISL_475083, EPI_ISL_475084, EPI_ISL_475165-EPI_ISL_475173, EPI_ISL_475238, EPI_ISL_475570, EPI_ISL_475571.

### Whole genome analysis

The nucleotide sequences of the whole genome of novel coronaviruses were analysed using Chromas 2.6.5 (Technelysium, Helensvale, Australia). Sequence homology was determined by using the BLASTn program (https://blast.ncbi.nlm.nih.gov/Blast.cgi). Multiple sequence alignment for the whole genome of Bangladeshi novel coronavirus strains and reference strains (Wuhan/WIV04/2019 and NC_045512/Wuhan-Hu-1) were conducted by using BioEdit 7.2.6 by using the ClustalW Multiple Alignment algorithm. Mutational analysis was performed for specific positions of novel coronavirus whole genome nucleotide sequences and peptide chains.

### Statistical analysis

All data were analysed using unbiased statistical approach. Spearman's rank correlation coefficient (*r*_s_) was determined between metrological parameters and COVID-19 cases and fatalities [14]. Further, Spearman's rank correlation coefficient (*r*_s_) was determined among environmental factors, host factors and mutation frequency of novel coronaviruses. The association between two variables can be defined using a monotonic function by using Spearman's rank correlation coefficient (*r*_s_). The coefficient equation can be written as
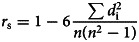


Here ‘*n*’ represented the number of alternatives, and ‘*d*_i_’ is the difference between the ranks of two parameters.

## Results

### Analysis of COVID-19 cases and fatalities

**Fig. 1.**
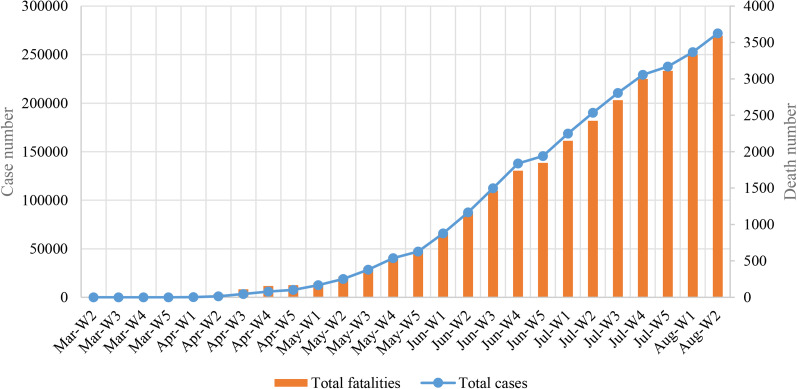
Total COVID-19 cases and fatalities in Bangladesh.

**Fig. 2.**
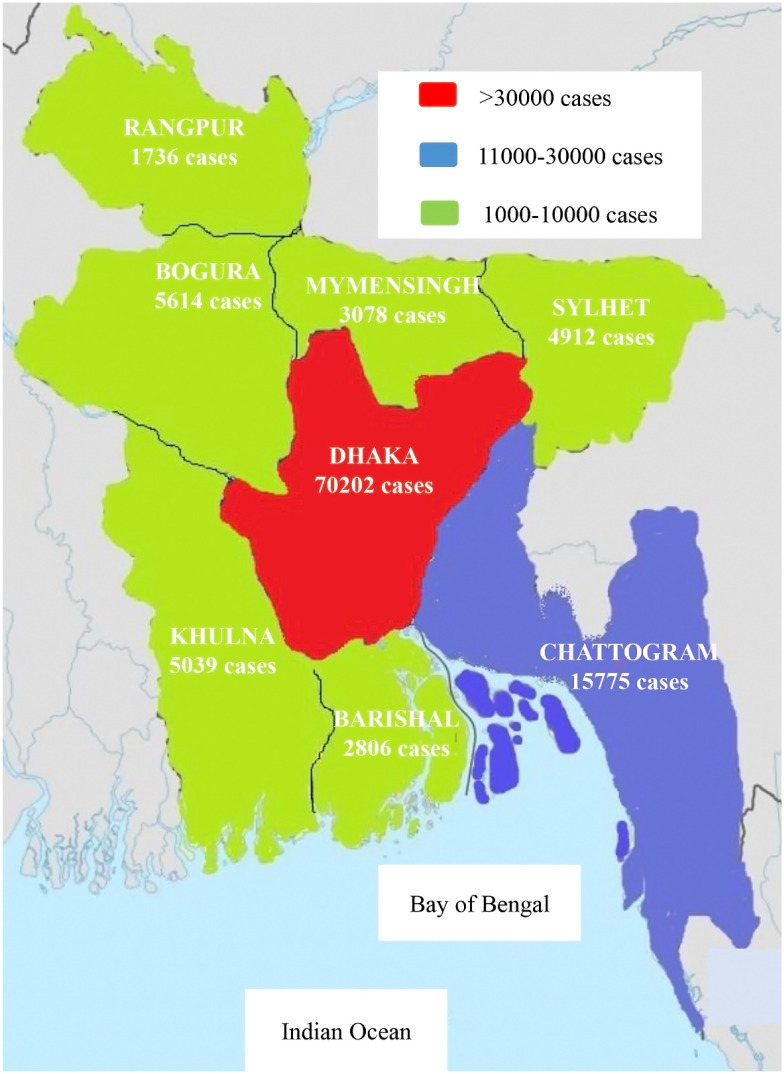
Map of the distribution of COVID-19 cases in eight different cities in Bangladesh

After 162 days from the detection of the first confirmed COVID-19 case in Bangladesh, the total cases had increased to 260 507 and fatalities to 3438 with 1.3% case fatality rate (Fig. 1). Most of the COVID-19 cases (*n* = 70 202) had been reported from Dhaka followed by Chattogram (*n* = 15 775) Bogura (*n* = 5614), Khulna (*n* = 5039), Sylhet (*n* = 4912) and Mymensingh (*n* = 3078), Barishal (*n* = 2806) and Rangpur (*n* = 1736), respectively (Table 1, Fig. 2). Among eight cities, Dhaka is the most populous and polluted city. The mobility of large number of workers is one of the major factors of rapid transmission of COVID-19 cases in Dhaka city.

**Table 1.** Distribution of COVID-19 cases in eight different cities in Bangladesh

Cities	Total COVID-19 cases
Dhaka	70 202
Chattogram	15 775
Bogura	5614
Khulna	5039
Sylhet	4912
Mymensingh	3078
Barishal	2806
Rangpur	1736

### Analysis of metrological parameters

**Fig. 3.**
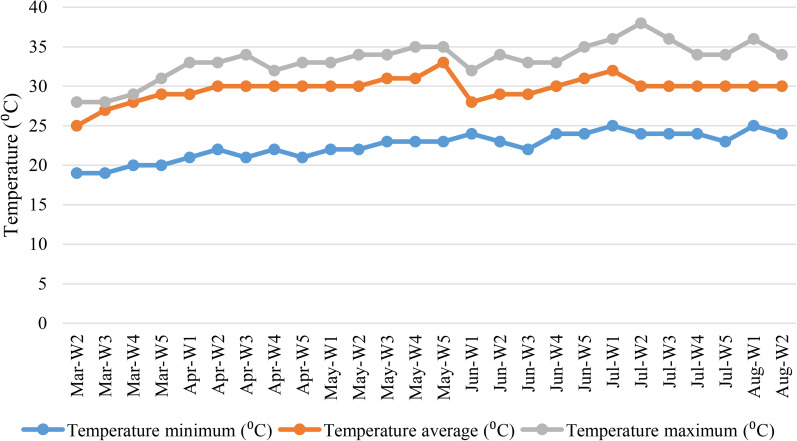
Weekly distribution of minimum temperature, maximum temperature and average temperature in Dhaka in Bangladesh.

Among metrological parameters, temperature is the most significant factor related with COVID-19 pandemic. The average of maximum temperature was 32 °C, and ranged from 28 °C to 38 °C in eight cities in Bangladesh. The average of minimum temperature was 21 °C, and ranged from 18 °C to 26 °C in eight cities in Bangladesh. The mean of average temperature was 27 °C, and ranged from 25 °C to 32 °C in Bangladesh during March 2020 to August 2020 (Fig. 3). The mean of minimum, maximum and average temperature varied ± 4 °C in eight cities. The lowest temperature was recorded in Rangpur (14 °C) on 14 March 2020 and the highest temperature was found in Dhaka (38 °C) on 02 July 2020.

**Fig. 4.**
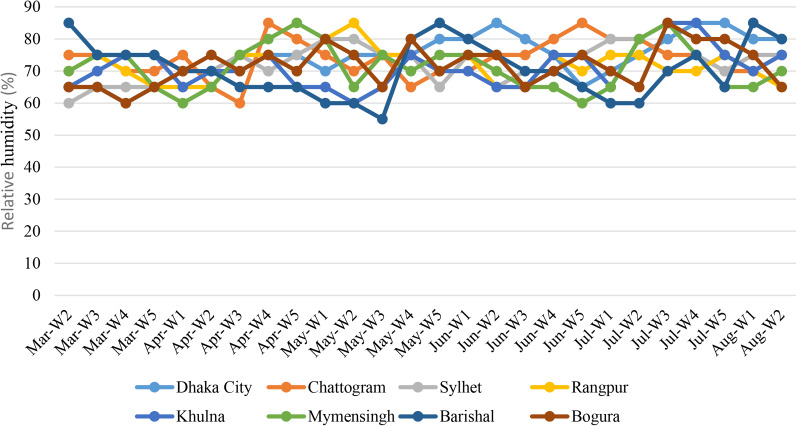
Average relative humidity of the cities per week in Bangladesh.

Relative humidity is another important metrological parameter associated with infectious diseases. The average relative humidity ranged from 55% to 85% in eight cities in Bangladesh. The highest average relative humidity was detected in Chattogram (78%), followed by Dhaka (76%), Rangpur (74%) and Khulna (73%), respectively (Fig. 4). The highest relative humidity (95%) in a single day was recorded from Barishal in 29 May 2020.

**Fig. 5.**
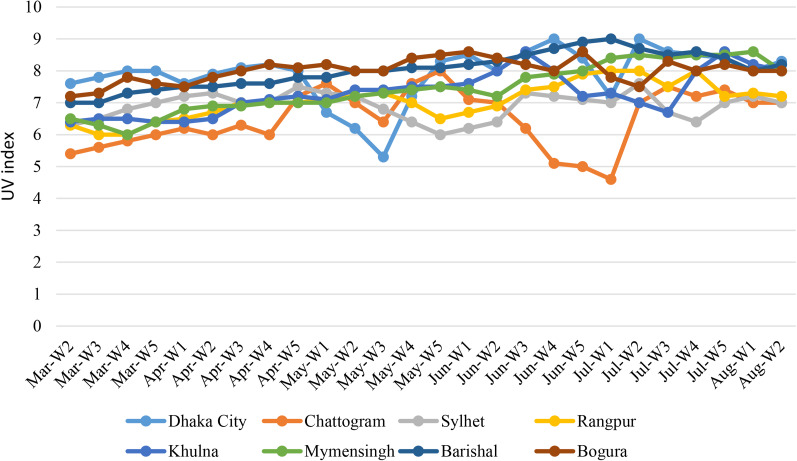
Weekly distribution of average UV index in the cities.

UV is an important metrological factor that affects the transmission and mutation frequency of novel coronaviruses. During this study, the average UV index was recorded between 6.5 and 8 in Bangladesh. The highest average UV index was recorded in Barishal and Bogura [7] followed by Dhaka (7.9), Mymensingh (7.4), Khulna (7.3), Rangpur (7), Sylhet (6.9) and Chattogram (6.5), respectively (Fig. 5). The highest UV index was recorded in Dhaka (9.8) on 24 June 2020.

**Fig. 6.**
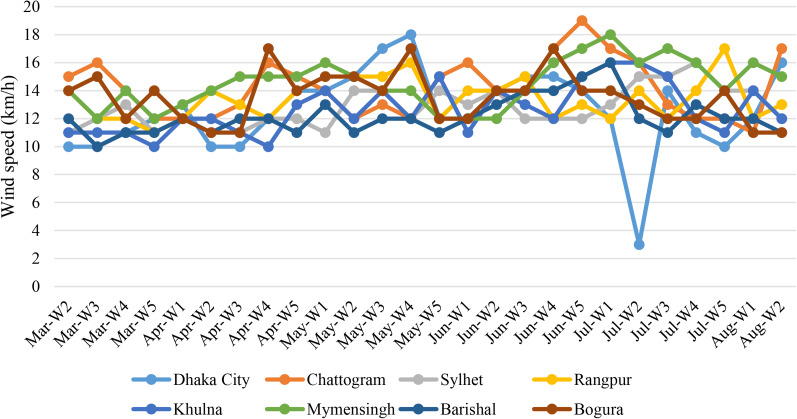
Weekly distribution of average wind speed in the cities.

Wind speed has direct effect on the spread of droplet nuclei containing virus particles. The average wind speed was recorded from 3 km/h to 19 km/h in this study. During this study, the highest average wind speed was recorded in Chattogram (17 km/h) followed by Bogura (16 km/h), Mymensingh (16 km/h), Khulna (15 km/h), Dhaka (14 km/h), Barishal (14 km/h), Rangpur (13 km/h) and Sylhet (11 km/h), respectively (Fig. 6).

Dhaka (46 997 person/km^2^) is the most populous city in the world with total population of 21 006 000, followed by Khulna (34 000 person/km^2^) with a total population of 1 122 000, Chattogram (19 800 person/km^2^) with a total population of 9 453 496, Sylhet (19 865 person/km^2^) with a total population of 526 412, Barishal (10 524 person/km^2^) with a total population of 385 093, Bogura (7763 person/km^2^) with a total population of 540 000, Mymensingh (5200 person/km^2^) with a total population of 476 543 and Rangpur (4167 person/km^2^) with a total population of 15 665 000, respectively.

### Spearman's correlation analysis between environmental data and COVID-19 pandemic

Spearman's correlation analysis between metrological and COVID-19 pandemic are presented in Table 2. As noted earlier, the six main environmental factors, minimum temperature, maximum temperature, average temperature, UV index, humidity, wind speed and total population as well as population density were considered in the study. Three time frames, namely on the day of the positive case, 7 days ago and 14 days ago of the positive cases were selected to evaluate six factors (minimum temperature, maximum temperature, average temperature, UV index, relative humidity and wind speed) First, the correlation between maximum temperature and the number of total cases in each city was evaluated. Similarly, the correlation between minimum temperature and total cases, average temperature and total cases were determined. Besides, correlation among three temperatures with total fatalities in each city were also determined. The average temperature on the day of the cases had the highest correlation (*r*_s_ = −0.675), followed by average temperature 7 days ago (*r*_s_ = −0.547), maximum temperature on the day (*r*_s_ = −0.512) and minimum temperature on the day (*r*_s_ = −0.486). Maximum temperature on the day had the highest correlation with total fatalities (*r*_s_ = −0.611). The correlation for both COVID-19 cases and fatalities are negative, which indicates that at lower temperature the number of cases and fatalities increases.

**Table 2.** Spearman's correlation coefficients of environmental factors and COVID-19 pandemic in Bangladesh

Environmental factors	Spearman's coefficient for COVID-19 cases	Spearman's coefficient for COVID-19 fatalities
Maximum temperature on the day	−0.512*	−0.611*
Maximum temperature 7 days ago	−0.411*	−0.478*
Maximum temperature 14 days ago	−0.379	−0.411
Minimum temperature on the day	−0.486**	−0.511**
Minimum temperature 7 days ago	−0.341	−0.374
Minimum temperature 14 days ago	−0.186	−0.277
Average temperature on the day	−0.675*	−0.598*
Average temperature 7 days ago	−0.547*	−0.475*
Average temperature 14 days ago	−0.411	−0.346
Relative humidity on the day	−0.399**	−0.412**
Relative humidity 7 days ago	−0.311	−0.300
Relative humidity 14 days ago	−0.249	−0.149
UV index on the day	−0.586*	−0.609*
UV index 7 days ago	−0.405	−0.499
UV index 14 days ago	−0.388**	−0.389**
Wind speed on the day	0.399**	0.247**
Wind speed 7 days ago	0.288*	0.189*
Wind speed 14 days ago	0.134	0.099
Population density	0.712*	0.678*
Total population	0.645*	0.578*

**, * stands for 1%, 5% level of significance.

Second, relative humidity on the day had the greater correlation with the number of fatalities than case number. The correlation between relative humidity and COVID-19 pandemic reduces with increasing time span. Third, the association between UV index and the number of cases is the highest on the day of the case. Similarly, the correlation between UV index and the number of fatalities was the highest on the day. The correlation of relative humidity and UV index with COVID-19 pandemic was also negative. Fourth, among environmental factors, the average wind speed on the day had the highest correlation with the number of cases. The higher the wind speed was, the more the number of cases and fatalities were. Finally, the total population and population density of a city were highly correlated with the number of cases and fatalities in the city. The population density had the highest correlation with cases (*r*_s_ = 0.712) and fatalities (*r*_s_ = 0.678) followed by the total population in every city.

### Spearman's correlation analysis between metrological parameters and novel coronavirus mutation frequency

The most important aspect of novel coronavirus is its ability to undergo frequent mutations and acquire the mutations through translation. Impact of six major metrological parameters, namely, minimum temperature, maximum temperature, UV index, relative humidity and rain as well as three host factors − coinfection, age and gender on mutation frequency were analysed in this study. The correlation was determined for the first 100 whole genomes of novel coronavirus from Bangladesh. Spearman's correlation coefficient was determined between metrological parameters and mutation frequency, as well as between host factors and mutation frequency (Table 3). Important mutational events, namely, common mutation at ORF1ab, rare mutation at ORF1ab, S (D614G), rare mutation at spike protein (S), first time mutation at S, common mutation at other structural proteins and rare mutations at other structural proteins were detected and included in this study. Most of the novel coronaviruses (87%) were from G clade in Bangladesh. Frequency of D614G had the highest correlation (*r*_s_ = 0.611) with average temperature but frequency of rare mutation at spike protein had the highest correlation (*r*_s_ = 0.658) with maximum temperature. Second, UV index was highly correlated with frequencies of all mutational events and the highest correlation was detected (*r*_s_ = 0.678) with rare mutation at ORF1ab. Third, relative humidity had highest correlation (*r*_s_ = 0.389) with frequency of D614G. Fourth, amount of rainfall was also strongly correlated with frequency of D614G. All of the metrological factors were positively related with the frequency of different mutations indicating increased temperature and UV index will favour origin of new mutations in the novel coronavirus. Finally, host factors-coinfection and gender variability were also positively correlated with mutation frequency. Coinfection had the highest association with common mutations at other structural proteins (*r*_s_ = 0.671) (Table 3). The age of the patients was negatively correlated with mutational events indicating that with an increase of age of the patients the mutational events decreased.

**Table 3.** Spearman's correlation coefficients of environmental factors, host factors and frequency of mutation in Bangladesh

Factors	Mutation site						
	Common at ORF 1ab	Rare at ORF 1ab	S (D614G)	Rare at S	First time at S	Common at other structural proteins	Rare at other structural proteins
Minimum temperature	0.449	0.412	0.411	0.354	0.114	0.456	0.389
Average temperature	0.654	0.598	0.611	0.547	0.451	0.446	0.510
Maximum temperature	0.588	0.499	0.364	0.658	0.398	0.384	0.460
UV index	0.643	0.678	0.577	0.504	0.614	0.514	0.460
Relative humidity	0.312	0.245	0.389	0.016	0.341	0.277	0.206
Rain	0.423	0.319	0.412	0.298	0.170	0.354	0.116
Coinfection	0.485	0.642	0.644	0.546	0.542	0.671	0.406
Gender	0.365	0.114	0.012	0.412	0.398	0.421	0.164
Age	−0.396	−0.315	−0.226	−0.194	−0.311	−0.105	−0.146

### Frequency of mutational events, age and gender distributions of the cases

**Fig. 7.**
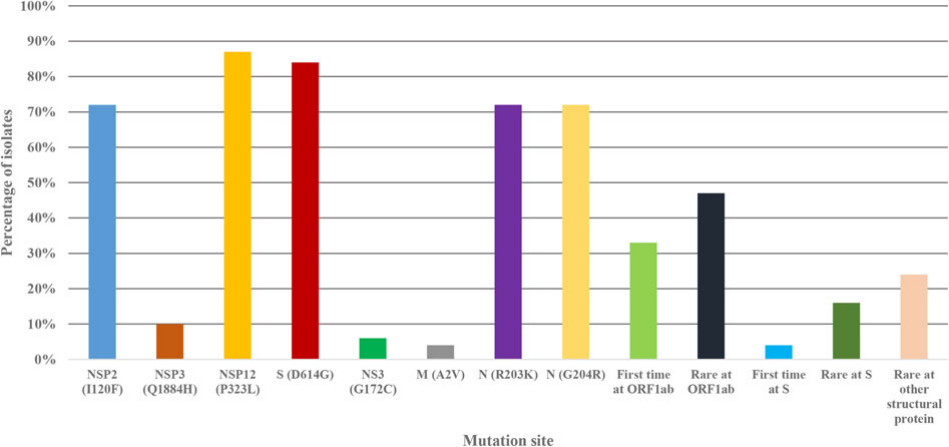
Frequency of mutations at different site in the Bangladeshi novel coronavirus genome.

After determining the first genome of the novel coronavirus, thousands of mutational events had occurred. Some of the mutations had made the virus more persistent, environmental resistant and more deadly. The first 100 genomes of the novel coronavirus were analysed in this study. Mutations were detected throughout the whole genome. The most common mutation at ORF1ab region were P323L (NSP12) (88%) and I120F (NSP2) (72%) (Fig. 7). The most important protein, S that is necessary for the successful binding of host cell receptor had also undergone mutations with high frequency. About 82% of the isolates from Bangladesh had D614G at S protein. Further, isolates with rare and first time mutation at S proteins loop region were detected in this study. Another two mutations, R203K and G204R at N protein were most common (73%) in the Bangladeshi novel coronaviruses (Fig. 7).

**Fig. 8.**
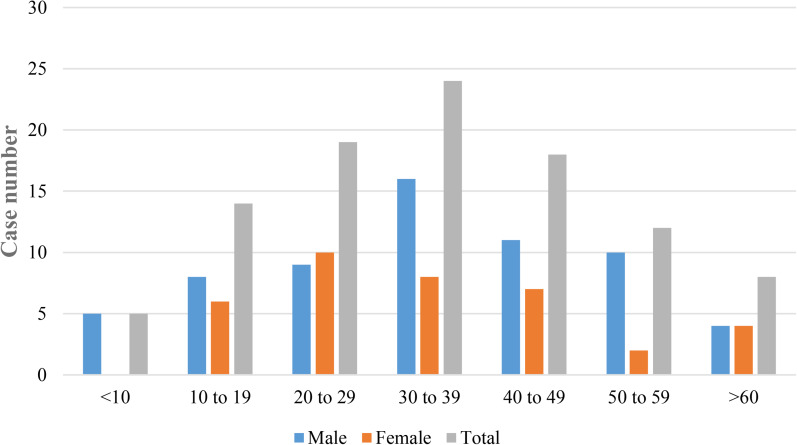
Age and gender distribution of the COVID-19 patients in Bangladesh.

Among 100 patients of this study, males were the predominant gender group (63%) followed by female patients (37%). Most of the cases (24%) were detected in the age group 30−39 years followed by 19% in 20−29 years and 18% in 40−49 years, respectively (Fig. 8).

## Discussion

With a moderate transmission rate, COVID-19 pandemic has transmitted throughout the entire world within very short time and continued to infect people [3, 4, 31, 32]. Various metrological parameters are correlated with COVID-19 pandemic [14, 20]. This study found very significant correlation between minimum temperature and COVID-19 cases, average temperature and COVID-19 cases, maximum temperature and COVID-19 cases, which is in good agreement with previous studies of COVID-19 correlation analysis in Wuhan, New York and Jakarta in 2020 [14, 20, 33]. However, previous studies had reported correlation between average temperature and COVID-19 cases, we detected stronger correlation between minimum temperature and COVID-19 cases (*r*_s_ = −0.486); between maximum temperature and COVID-19 cases (*r*_s_ = −0.512); between average temperature and COVID-9 cases (*r*_s_ = −0.675) [33, 34]. Four time frames were used to determine the correlation between metrological parameters and COVID-19 cases in a recent study, but we considered three time frames for both the cases and fatalities [34]. Tosepu *et al*. [33] did not report any significant correlation between other metrological parameters, namely, relative humidity, rainfall, wind speed, precipitation and COVID-19 pandemic while Şahin [34] reported significant correlation with these metrological parameters and COVID-19 cases in Turkey. This study reported stronger correlation among these metrological parameters and COVID-19 cases/fatalities than previous study [34]. None of the previous studies reported the correlation between UV index and COVID-19 pandemic but we detected strong correlation between UV index and cases (*r*_s_ = −0.586)/ fatalities (*r*_s_ = −0.609) [33, 34]. While considering time frames, previous study detected significant correlations between metrological parameters and COVID-19 cases on the day of the cases except wind velocity [34]. In this study, the correlations between metrological factors and cases/fatalities were also the highest on the day of cases/fatalities including wind velocity.

Previous studies have reported significant correlation of coronaviruses infection with metrological parameters [16, 35, 36]. High temperature can reduce the survival period of coronaviruses in droplet nuclei [37]. Both laboratory and environmental studies have found that above 38 °C the viability of coronaviruses are reduced significantly [36, 37]. Another study reported that temperature and relative humidity have a notable effect on the *r*_s_ value of COVID-19 cases in China [32]. Further, recent studies have reported about significant correlation of environmental factors and COVID-19 in Wuhan, New York, Turkey and Jakarta, respectively [14, 20, 33, 34]. They specifically reported correlation of temperature and humidity with COVID-19 pandemic. In this study, significant and stronger correlations between metrological factors and COVID-19 cases/fatalities were detected which is in good agreement with previous studies. Previous studies had reported the effects of UV index on the viability of coronaviruses, namely, SARS-CoV and MERS-CoV [38, 39]. This study has also reported significant correlation between UV index and COVID-19 cases/fatalities which is in good agreement with previous studies [38, 39].

Şahin [34] detected a strong correlation (*r*_s_ = 0.687) between total population and COVID-19 cases in Turkey. However, this study includes both the total population and population density for COVID-19 cases and fatalities. This study reported stronger correlation between total population and cases (*r*_s_ = 0.645) /fatalities (*r*_s_ = 0.578); between population density and cases (*r*_s_ = 0.712) /fatalities (*r*_s_ = 0.678) than previous study [34].

Mutational events are most important aspects for novel coronavirus infection, transmission and persistence in the host cells. Mutations at spike proteins can create difficulties to develop effective vaccine or therapeutics against novel coronaviruses [9, 29, 30]. While analysing the first 100 full genome sequences of novel coronaviruses in Bangladesh, this study detected a diverse frequency of mutations at various sites. These mutational events can lead to persistent, deadly, immune-tolerant and environment-tolerant isolates. Previous studies have already addressed the effects of mutations on COVID-19 pandemic [40, 41]. This study reported common mutations at ORF1ab-P323L (NSP12) (88%) and I120F (NSP2) (72%); S-D614G (82%), N-R203K (73%) and G204R (73%) and other structural proteins with high frequency. Of note, significant rare and new mutations were also detected at ORF1ab (48% and 33%) S (17% and 4%) and N (20%) sites with high frequency. Frequency of mutational events were higher than previous reports [40, 41]. We analysed both gender distribution and age distribution of the cases. This study reported the most common patients being male (63%) and of age group 30−39 years was the most infected (24%), followed by 20−29 years (19%) and 40−49 years (18%), respectively. Both the gender and age distribution of cases in Bangladesh were different from previous studies [1, 7].

This study determined the correlation between metrological parameters and mutational frequency of novel coronaviruses for the first time. This study included both metrological parameters and host factors to determine correlation with frequency of mutations. Studies have found significant effects of environmental factors and UV radiation on the frequency of mutation of influenza virus and Newcastle disease virus [42, 43]. This study reported strong correlation between average temperature and mutation frequency at ORF1ab-common mutation (*r*_s_ = 0.654), rare mutation (*r*_s_ = 0.598) and at S- D614G (*r*_s_ = 0.611); between maximum temperature and rare mutation at S (*r*_s_ = 0.658). Among other metrological factors, UV index had the highest correlation with frequency of mutations at every site in the genome. Among host factors, coinfections had strong correlation with frequency of mutations at ORF1ab- common mutation (*r*_s_ = 0.485), rare mutation (*r*_s_ = 0.642), at S-D614G (*r*_s_ = 0.644) and common mutation at other protein (*r*_s_ = 0.671). Both age and gender had significant correlation with frequency of mutations at many sites of the genome. As of 18 August 2020 no other study has described the correlation between metrological parameters/host factors and frequency of mutations in novel coronaviruses. However, for other viruses like influenza and Newcastle disease viruses there are reports of environmental and host factors association with the virus mutations [42, 43].

This study has described the highest correlation between metrological parameters and COVID-19 cases; strong correlation between metrological parameters and COVID-19 fatalities; first time correlation between metrological factors and frequency of mutations in novel coronaviruses; first time correlation between host factors and frequency of mutation. This study also provided the mutation frequency at different sites of novel coronaviruses. A complete picture on the effect of metrological parameters on COVID-19 pandemic and novel coronavirus mutation frequency has been depicted in this study. This study will work as a baseline for the future studies focusing on the environmental and host factors affecting the COVID-19 pandemic. Further, this study will work as a guideline for future studies by containing important information on environmental and host factors impact on mutations of coronaviruses.

Other factors like duration of lockdown, mobility of huge number of workers, social and religious gatherings, not using masks, movement of people during vacations, lack of proper detection of COVID-19 patients etc. are significantly affecting the pandemic. If direct contact is not avoided, social distance and personal hygiene are not maintained, environmental factors cannot control the COVID-19 pandemic alone. This study describes high frequency of common and rare mutations in the novel coronavirus genome in Bangladesh. Circulation of these mutants will certainly increase the duration of the pandemic and reduce the effectiveness of vaccine or therapeutics in future. The main limitation of this study is the variation of case number. The actual case and fatality number may vary slightly due to the lack of complete diagnosis of the population. In future, studies including more isolates of viruses, large number of clinical data and environmental data of longer periods can predict more accurate effects of various factors on COVID-19.

## Conclusion

To the best of our knowledge, this is the first study reporting correlation of environmental factors with COVID-19 pandemic in three time frames in Bangladesh with temperature about 27 °C. The strongest correlations between metrological factors and COVID-19 cases/fatalities were specified on the day of cases/fatalities. The highest correlation was detected between population density and cases followed by total population and cases indicating the mobility and crowd are actively increasing the cases and fatalities. For the first time, this study describes the effects of metrological parameters on the frequency of mutation at different sites in novel coronavirus. Both temperature and UV index had strong effects on different mutation events. Among host factors, coinfection also affected different mutations strongly. Including COVID-19 cases, fatalities, mutations, mutation frequency and clinical data this study provides a complete picture of the COVID-19 pandemics and environmental effects on this pandemic. This study will provide useful implications about COVID-19 pandemic and both for policy makers and public to take decision to reduce the health burden of this outbreak.

## Data Availability

Restrictions apply to the availability to the data that support the findings of this study.
